# *Synedrella nodiflora* Extract Depresses Excitatory Synaptic Transmission and Chemically-Induced *In Vitro* Seizures in the Rat Hippocampus

**DOI:** 10.3389/fphar.2021.610025

**Published:** 2021-03-08

**Authors:** Patrick Amoateng, Thomas A. Tagoe, Thomas K. Karikari, Kennedy K. E Kukuia, Dorcas Osei-Safo, Eric Woode, Bruno G. Frenguelli, Samuel B. Kombian

**Affiliations:** ^1^ Department of Pharmacology and Toxicology, School of Pharmacy, College of Health Sciences, University of Ghana, Accra, Ghana; ^2^ Department of Physiology, UG Medical School, College of Health Sciences, University of Ghana, Accra, Ghana; ^3^ Department of Psychiatry and Neurochemistry, Institute of Neuroscience and Physiology, Sahlgrenska Academy, University of Gothenburg, Gothenburg, Sweden; ^4^ Department of Medical Pharmacology, UG Medical School, College of Health Sciences, University of Ghana, Accra, Ghana; ^5^ Department of Chemistry, School of Physical and Mathematical Sciences, College of Basic and Applied Sciences, University of Ghana, Accra, Ghana; ^6^ Department of Pharmacology and Toxicology, School of Pharmacy, University of Health and Allied Sciences, Ho, Ghana; ^7^ School of Life Sciences, University of Warwick, Coventry, United Kingdom; ^8^ Department of Pharmacology and Therapeutics, Faculty of Pharmacy, Health Science Center, Kuwait University, Safat, Kuwait; ^9^ Department of Pharmacology and Toxicology, School of Medicine and Medical Sciences, University for Development Studies, Tamale, Ghana

**Keywords:** hippocampal slices, field excitatory postsynaptic potentials, adenosine, SNE, seizure

## Abstract

Extracts of the tropical Cinderella plant *Synedrella nodiflora* are used traditionally to manage convulsive conditions in the West African sub-region. This study sought to determine the neuronal basis of the effectiveness of these plant extracts to suppress seizure activity. Using the hippocampal slice preparation from rats, the ability of the extract to depress excitatory synaptic transmission and *in vitro* seizure activity were investigated. Bath perfusion of the hydro-ethanolic extract of *Synedrella nodiflora* (SNE) caused a concentration-dependent depression of evoked field excitatory postsynaptic potentials (fEPSPs) recorded extracellularly in the CA1 region of the hippocampus with maximal depression of about 80% and an estimated IC_50_ of 0.06 mg/ml. The SNE-induced fEPSP depression was accompanied by an increase in paired pulse facilitation. The fEPSP depression only recovered partially after 20 min washing out. The effect of SNE was not stimulus dependent as it was present even in the absence of synaptic stimulation. Furthermore, it did not show desensitization as repeat application after 10 min washout produced the same level of fEPSP depression as the first application. The SNE effect on fEPSPs was not via adenosine release as it was neither blocked nor reversed by 8-CPT, an adenosine A_1_ receptor antagonist. In addition, SNE depressed *in vitro* seizures induced by zero Mg^2+^ and high K^+^ -containing artificial cerebrospinal fluid (aCSF) in a concentration-dependent manner. The results show that SNE depresses fEPSPs and spontaneous bursting activity in hippocampal neurons that may underlie its ability to abort convulsive activity in persons with epilepsy.

## Introduction

The use of plants as a source of drugs is long-standing and widespread in developing countries (Rates, 2001). It is a dominant source of therapy for many diseases in Ghana (Abel and Busia, 2005; Van Andel et al., 2012; Amoateng et al., 2018a). Plants have frequently also been the source of lead compounds, from which newer and effective drugs can be developed (Soh and Benoit-Vical, 2007; Amoateng et al., 2012; Van Andel et al., 2012). A number of plant products have traditionally been used in the management of epilepsy in Ghana (Mshana et al., 2000; Adjei, 2017; Amoateng et al., 2018b) and some of these have been investigated on animal models of seizures with promising efficacy and minimal side effects (; Stafford et al., 2008; Woode, et al., 2011a; Woode, et al., 2011b; Amoateng et al., 2012; Mante et al., 2013; Kukuia et al., 2016).

One of such plants is *Synedrella nodiflora,* a weed which grows abundantly in Ghana mainly in water-logged and shady areas. This plant has a long history of use in Ghana for the management of epilepsy (Mshana et al., 2000). Previous studies have demonstrated anticonvulsant (Amoateng et al., 2012), analgesic (Woode et al., 2009; Amoateng et al., 2015) and antioxidant effects (Amoateng et al., 2011) of the hydro-ethanolic extract of this plant, validating its traditional medicinal use. In addition, acute, sub-acute and sub-chronic toxicological investigations of the extract reveal minimal or no observable toxicity in rats (Adjei, et al., 2014a; Adjei, et al., 2014b; Amoateng et al., 2016). The *in vivo* models used for the anticonvulsant studies were chemically and electrically induced seizures in rats and mice. Although *in vivo* studies provide useful functional results, they generally have the disadvantage that, it is difficult to elucidate underlying cellular mechanism(s) of the anticonvulsant effects. In order to more completely characterize and understand how this plant’s extracts produce the functional outcomes described above, it is necessary to examine their effects at the cellular level, which affords more detailed pharmacological investigations and analysis. The effects of potential anticonvulsant agents on neurons and on appropriate *in vitro* seizure models have the advantage of allowing mechanistic screening of the anticonvulsant agents (Scharfman and Schwartzkroin, 1990; Kupferberg, 2001; Kreir et al., 2018). These models involve the use of brain slices of region with low seizure threshold such as the hippocampal CA1 or CA3 areas where seizures can be induced either electrically or chemically (Johnston and Brown, 1986; Wong, 2011). Chemical induction of seizures can be achieved by simple manipulations of altering ionic composition of the artificial cerebrospinal fluid (aCSF) such as lowering calcium concentration (Feng and Durand, 2003; Vogel and Vogel, 2013) or removing Mg^2+^ (Anderson et al., 1986; Gean and Shinnick-Gallagher, 1988; Köhling et al., 2001; Vogel and Vogel, 2013). Other forms of chemically induced seizures include, blocking of GABA_A_ receptors or their channels in the presence of elevated K^+^ (Hablitz and Heinemann, 1987; Traynelis and Dingledine, 1988; Khazipov et al., 2015), or activating glutamate receptors e.g., with kainic acid (Lothman and Collins, 1981). On the other hand, electrically induced seizures can produced by patterned electrical stimulation such as the stimulus train-induced bursts (STIBs) (Stasheff et al., 1985; Tian et al., 1991; Klapstein and Colmers, 1997; Huberfeld et al., 2015). In this study, we investigated the actions of the hydro-ethanolic extract of *Synedrella nodiflora* (SNE) on evoked excitatory field potentials recorded in the CA1 area of the hippocampus and on chemically induced seizures in hippocampal slices. We report here that SNE depressed the amplitude of fEPSPs and chemically induced spontaneous burst frequency and amplitude in a concentration-dependent manner. These effects likely involved presynaptic mechanisms but were not mediated by adenosine, a known presynaptic modulator.

## Materials and Methods

### Plant Extract Preparation

Plant samples were collected from the Botanical Gardens, University of Ghana, Accra in August 2016. The plant was identified and authenticated at the Ghana Herbarium, Department of Plant and Environmental Biology, University of Ghana, Legon, Accra after which a voucher specimen (PA03/UGSOP/GH16) was prepared and kept at the same department. The ethanolic extract was prepared as previously described (Amoateng et al., 2017). Briefly, the plant stems with leaves were air-dried for seven days, ground into powder, and cold-macerated with 70% v/v ethanol in water. The resultant hydro-ethanolic extract was evaporated using a rotary evaporator (Buchi Rotavapor® R-300, Flawil, Switzerland) under reduced pressure to eliminate traces of ethanol. The aqueous portion was frozen at −20°C and lyophilized (Bench-top Freeze Dryer, Labfreez Instruments Co., Ltd, Beijing, China). The percentage yield of dried SNE was calculated (10% w/w) and the extract labeled (as SNE) and stored in a refrigerator at 4–8°C.

### High Performance Liquid Chromatography

An HPLC fingerprint was obtained as previously described (Amoateng et al., 2017). Briefly, a Perkin Elmer Flexar HPLC (high performance liquid chromatography), fitted with a PDA detector and a manual injector was used. The components of SNE were separated on a µBondapak C18 Column (150 × 4.6 mm, 3 µm) with mobile phase 0.1% formic acid (A) and Methanol (B). The gradient elution commenced with 100% for 10 min and then moved to 50% in 40 min. It was kept at 50% for another 10 min and returned to 100% in 2 min, making a total run time of 62 min. The flow rate was 1 ml/min and the sample injection were 100 µl (0.108 g in 1:4 methanol-H_2_O mixture). The wavelength was set at 315 nm.

### Chemicals and Drugs

SNE was prepared as 20 mg/ml stock solution in an appropriate artificial cerebrospinal fluid (aCSF, composition described below), centrifuged at 1,792 g (relative centrifugal force) for 10 min, the supernatant collected, aliquoted and stored frozen at −20°C. 8-cyclopentyltheophylline (8-CPT) (Sigma-Aldrich, Poole, Dorset, United Kingdom) was prepared as 2 mm stock solutions and frozen until use. On the day of use, the stock solutions were thawed, and aliquots diluted with aCSF to the desired concentrations. Each stock preparation was used within 2 weeks and any remnants were discarded.

### Brain Slice Preparation

Sagittal hippocampal slices (350–400 μm) were prepared from 15 to 20-day-old Sprague-Dawley rats of either sex. The animals were killed by cervical dislocation in accordance with the United Kingdom Government Animals (Scientific Procedures) Act 1986, and with the approval of the University of Warwick’s Animal Welfare and Ethical Review Board. The animals were then decapitated, and the whole brain rapidly removed, and cerebellum and olfactory bulb discarded. Slices were prepared, from separated hemispheres, glued on their lateral aspects to the cutting chuck of a Microm HM 650 V microslicer (Carl Zeiss, Welwyn Garden City, United Kingdom) in ice-cold (∼4°C) high Mg^2+^, low Ca^2+^ artificial cerebrospinal fluid (aCSF), composed of (mM): 127 NaCl, 1.9 KCl, 8 MgCl_2_, 0.5 CaCl_2_, 1.2 KH_2_PO_4_, 26 NaHCO_3_, 10 D-glucose. Slices were stored in standard aCSF (containing all the above but with 1 mm MgCl_2_, and 2 mm CaCl_2_) at 35°C for at least 1 h (bubbled with 95% O_2_ and 5% CO_2_) before recording.

### Extracellular Recording From Slices

An individual slice was transferred to a recording chamber where it was suspended on nylon mesh grid, completely submerged in standard aCSF and perfused at 6 ml/min, to allow all round perfusion and thus reducing the risk of hypoxia. All perfusion solutions were bubbled with 95% O_2_ and 5% CO_2_ and maintained at around 32°C. All tubing used had low gas permeability (Tygon; Fisher Scientific, Loughborough, United Kingdom). In order to record synaptic responses, a concentric tungsten bipolar metal stimulating electrode was placed in *stratum radiatum* in the CA1 region to stimulate afferent Schaffer collaterals/commissural fibers, at 15 s intervals (100 μs pulses). A single aCSF-filled glass microelectrode was positioned in the CA1 dendritic region to record evoked field excitatory postsynaptic potentials (fEPSPs; filtered at1 Hz-3 kHz). WinLTP® software was used to control stimulus parameters and acquisition, as well as to record and analyze the slope of fEPSPs (Anderson et al., 2012).

### Experiments on Synaptic Transmission

After ensuring a stable baseline fEPSP recording in standard aCSF (∼20 min), the medium was changed to increasing concentrations of SNE (0.01, 0.1, and 1.0 mg/ml). The application was done in a continuous, cumulative manner except where indicated otherwise. Following application of the last concentration of SNE, the medium was changed back to standard aCSF. For determination of paired pulse facilitation (PPF), the afferents were stimulated twice in succession (50 ms interpulse interval) and paired pulse ratios (PPR) were determined (pulse 2/pulse 1) before and after application of SNE. In another set of experiments to study if SNE caused desensitization, SNE (1 mg/ml) was applied without any stimulation for 10 min, after establishing the baseline. Then the tissue was stimulated and SNE washed out to recovery, followed by reapplication of SNE under stimulation. To investigate the possible interaction of SNE with adenosine receptors, tissues were pretreated with the adenosine A_1_ receptor antagonist 8-cyclopentyltheophylline (8-CPT, 4 µm) for 10 min and subsequently with different concentrations of SNE in the continued presence of 8-CPT (4 µm). In another set of experiments, all concentrations of SNE up to the maximum of 1 mg/ml were applied and at their peak effect, 8-CPT (4 µm) was applied in their presence to investigate possible reversal of the synaptic depression caused by SNE. IC_50_ and paired pulse ratio (PPR) data were all expressed as mean ± SEM.

### Seizure Protocol

After establishing a stable baseline of fEPSPs in standard aCSF (∼5 min), stimulation was halted, and the perfusion medium was replaced with Mg^2+^-free, high KCl (4.9 mM) aCSF in order to induce seizure activity. After 3-4 successive seizure bursts, the medium was switched to apply increasing concentrations of SNE (0.01, 0.1, and 1.0 mg/ml in Mg^2+^-free medium) in a cumulative fashion. Seizure activity was reversed by reperfusion with normal aCSF. Electrophysiological recordings were made using Spike2^®^ software. Burst frequency and size were determined in control and at the peak of SNE application. All data are expressed as mean ± SEM.

### Data Acquisition, Analysis and Statistics

Drugs/extract were administered for 10 min after control periods were established. All data are expressed as mean ± SEM and *n* = number of slices. Statistical significance of all measures was determined using Student’s t-test/ANOVA where appropriate (paired or unpaired for *t*-test, and one-or-two way ANOVA) and considered significant at *p* ≤ 0.05 using GraphPad Prism for Windows version 5.0 (GraphPad Software, San Diego, CA, United States). fEPSP amplitudes were normalized by taking the mean of 4-5 responses prior to drug application and dividing the rest of the values by this mean. The values were used for average plots and bar graphs. All graphical representations, were done using SigmaPlot® (Systat Software Inc., San Jose, CA, United States), Origin® (Originlab Corporation, MA, United States) and CorelDraw® (Corel Corporation, Ottawa, ON, Canada).

## Results

### High Performance Liquid Chromatography

**FIGURE 1:**
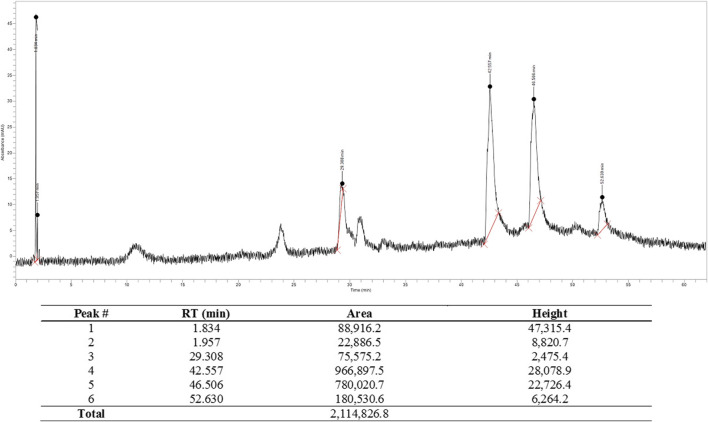
High performance liquid chromatography chromatogram of SNE monitored at 315 nm.

From the chromatogram (Figure 1) two major components were seen at retention times 42.56 and 46.51 min. The percentage composition was determined to be 45.72 and 36.88% respectively, using the areas under the curve (AUCs) as previously reported (Amoateng et al., 2017).

### SNE Depressess Synaptic Transmission by a Presynaptic Mechanism

**FIGURE 2:**
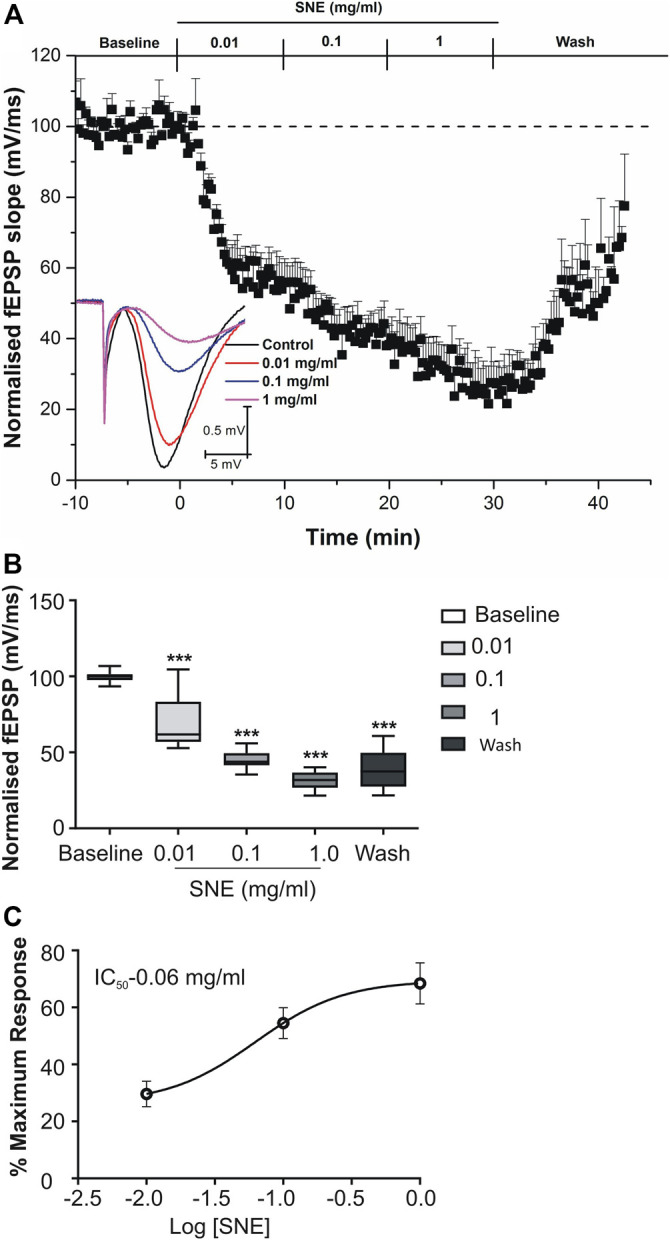
**(A)** Effect of SNE (0.01, 0.1 and 1.0 mg/ml) on slope of fEPSPs in rat hippocampal slices after a stable baseline perfused with normal aCSF. **(B)** The bar graph represents the total effect (calculated as AUCs from the graph above). **(C)** Concentration-response curve from SNE (0.01, 0.1, and 1.0 mg/ml) on slope of fEPSPs. Data is mean ± SEM (n = 5). ***p < 0.001 compared with baseline, one-way ANOVA followed by a Dunnett’s multiple comparison test.

**FIGURE 3:**
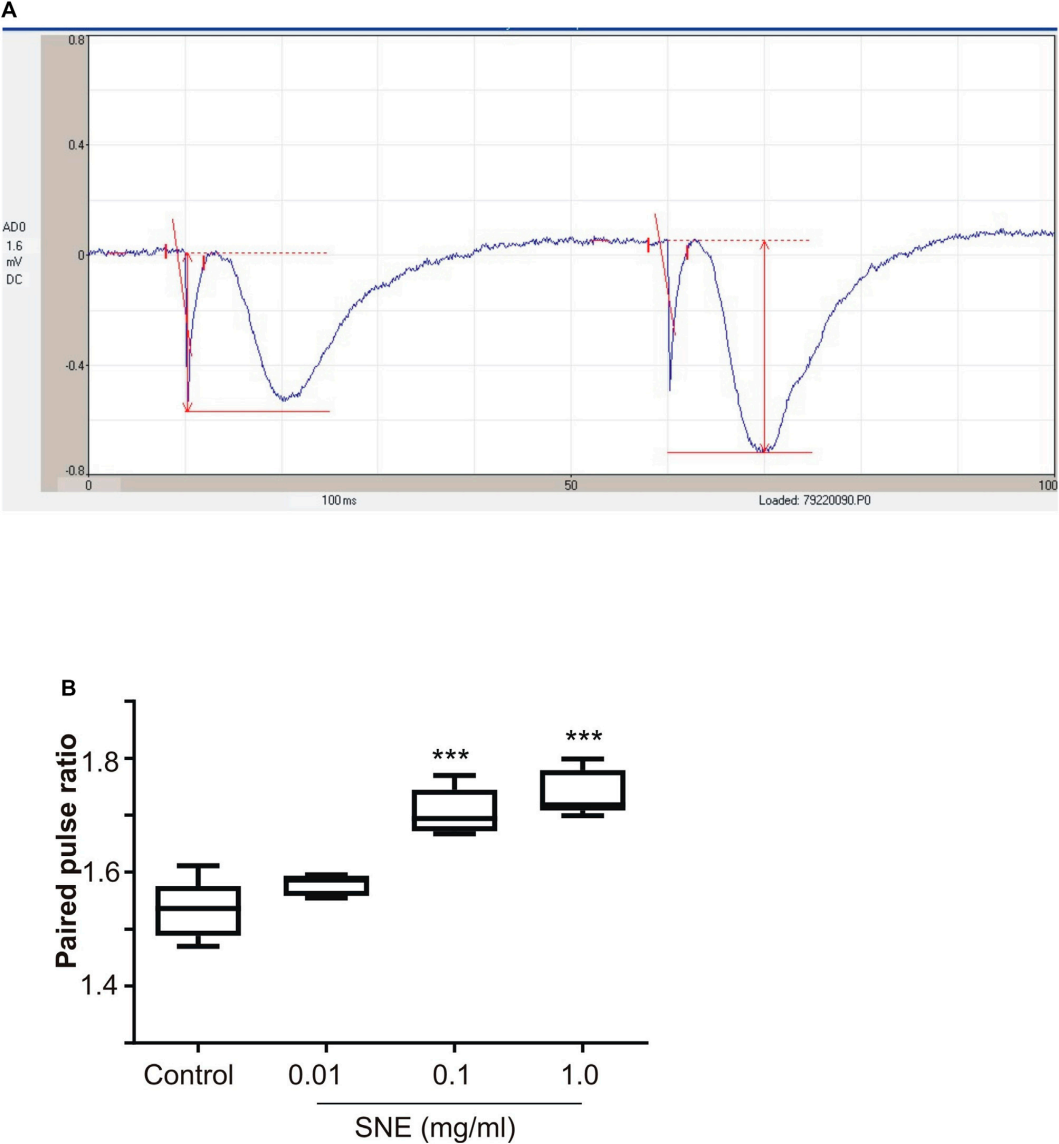
**(A)** A typical recording of paired fEPSP showing pair-pulse facilitation **(B)** Effect of SNE (0.01, 0.1, and 1.0 mg/ml) on paired pulse ratio in rat hippocampal slices after a stable baseline perfused with normal aCSF. Data is mean ± SEM (n = 5). ***p < 0.001 compared with baseline, one-way ANOVA followed by a Dunnett’s multiple comparison test.

**FIGURE 4:**
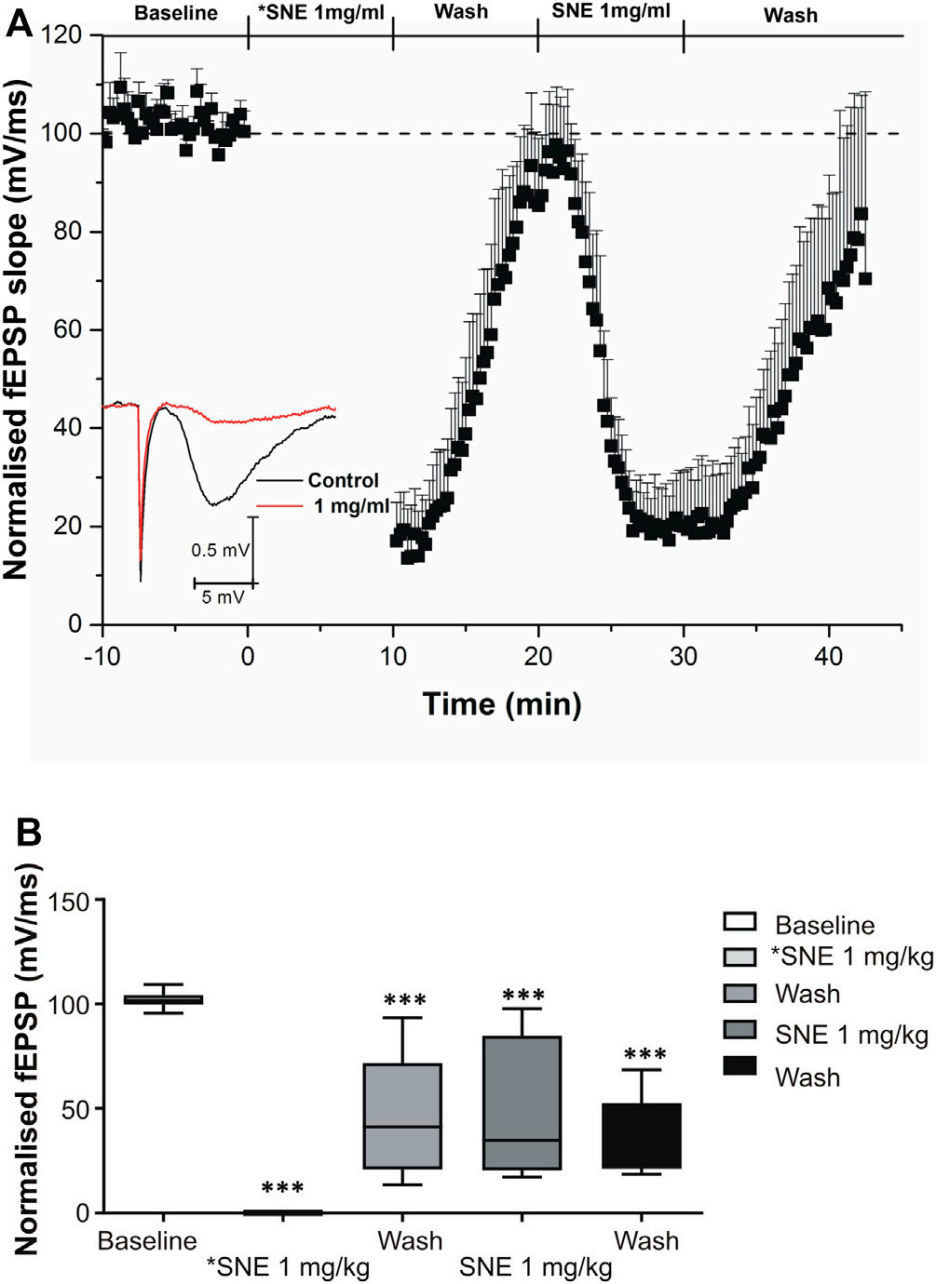
**(A)** Effect of SNE (1 mg/ml) on the slope fEPSPs without stimulation (*SNE 1.0 mg/ml) and with stimulation (SNE 1.0 mg/ml). **(B)** The box and whiskers graph is the total effect calculated form the AUCs of the graph above it. Data is mean ± SEM (n = 5). ***p < 0.001 compared with baseline, one-way ANOVA followed by a Dunnett’s multiple comparison test.

**FIGURE 5:**
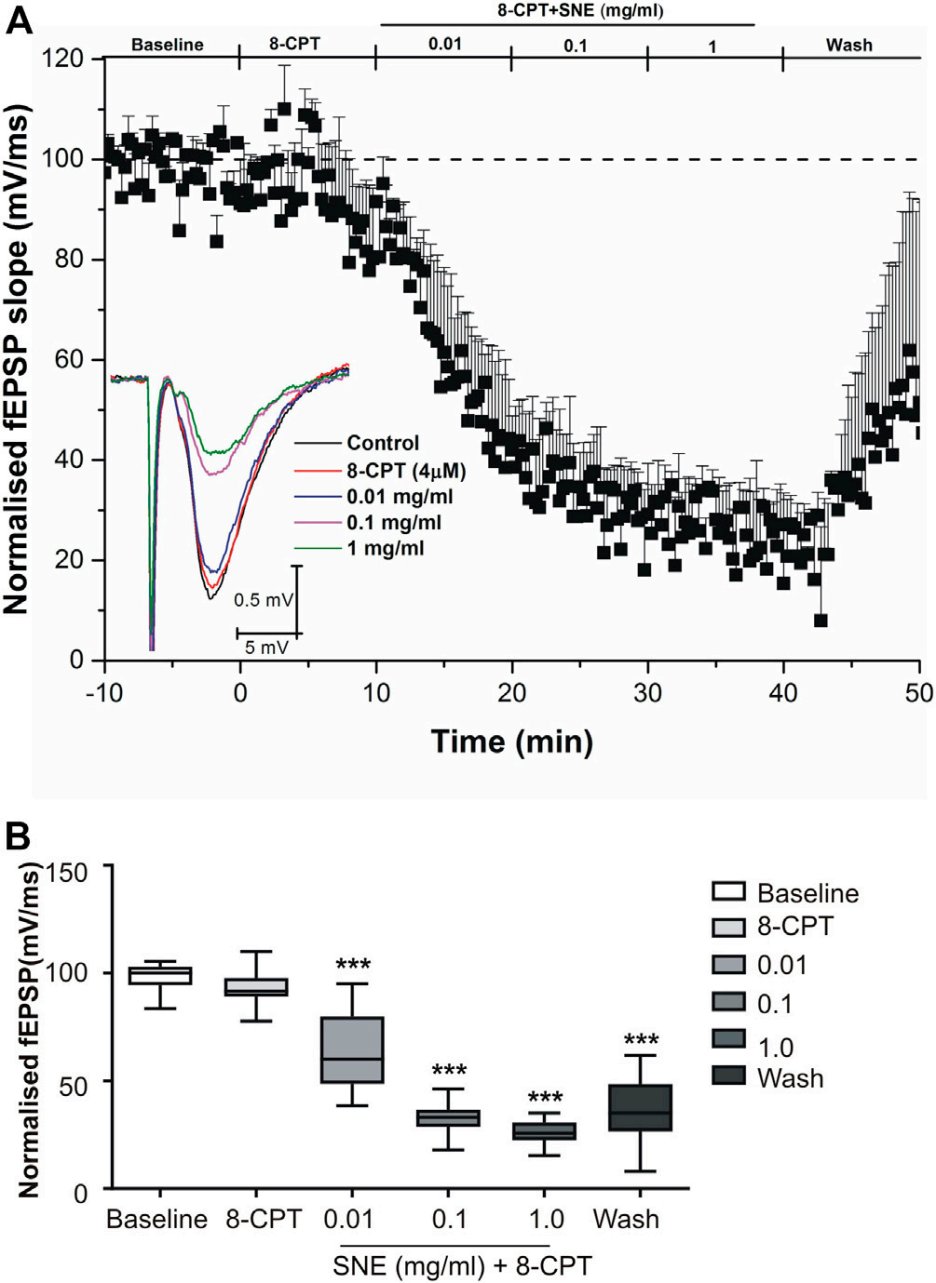
**(A)** Administration of 8-CPT (4 µM) before SNE (0.01, 0.1 and 1.0 mg/ml) on slope of fEPSPs in rat hippocampal slices after a stable baseline perfused with normal aCSF. **(B)** The box and whiskers graph is the total effect calculated form the AUCs of the graph above it. Data is mean ± SEM (n = 3). ***p < 0.001 compared with baseline, one-way ANOVA followed by a Dunnett’s multiple comparison test.

**FIGURE 6:**
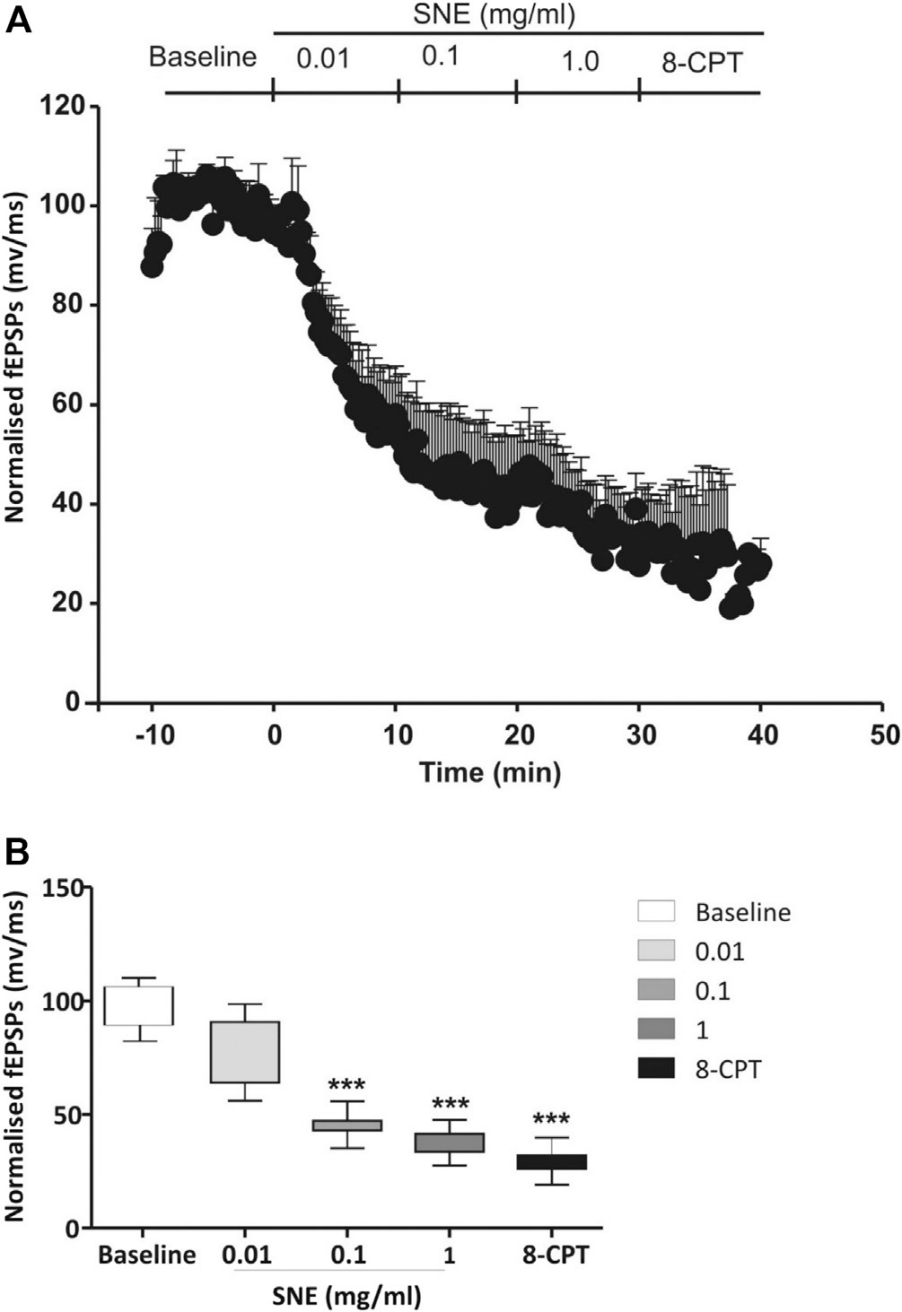
**(A)** Administration of 8-CPT (4 µM) after SNE (0.01, 0.1 and 1.0 mg/ml) on slope of fEPSPs in rat hippocampal slices after a stable baseline perfused with normal aCSF. **(B)** The box and whiskers graph is the total effect calculated form the AUCs of the graph above it. Data is mean ± SEM (n = 3). ***p < 0.001 compared with baseline, one-way ANOVA followed by a Dunnett’s multiple comparison test.

SNE depressed fEPSPs in a concentration-dependent manner with an estimated IC_50_ of 0.06 mg/ml (Figures 2, 3A). Approximately 50% of the depressant effect of SNE was recovered after 20 min of washing out SNE. Under control conditions the paired pulse ratio (slope of fEPSP 2/slope of fEPSP1) was above 1 (1.5 ± 0.5) indicating paired-pulse facilitation (PPF). The paired-pulse ratio increased significantly above this control level of facilitation in the presence of 0.1 and 1.0 mg/ml SNE (Figure 3B, *p* < 0.05). The depressant effect of SNE (1 mg/ml) was not dependent on stimulation of the hippocampal slices as the response was depressed to similar levels in the absence of stimulation (Figure 4). Furthermore, repeat application of SNE did not lead to an enhanced depressant effect compared to the single application, 80.5 ± 6.5% vs. 82.7 ± 8.5% (Figure 4A,B, *p* > 0.05). Pre-treatment of hippocampal slices with 8-CPT (4 μm) did not prevent or block the ability of any of the tested doses of SNE to depress fEPSPs (Figure 5). Furthermore, in another set of slices, when SNE had induced peak fEPSP depression at the highest concentration tested, subsequent application of 4 μm 8-CPT did not reverse the synaptic depression induced by SNE (Figure 6).

### SNE Suppresses Seizure-Like Activity

**FIGURE 7:**
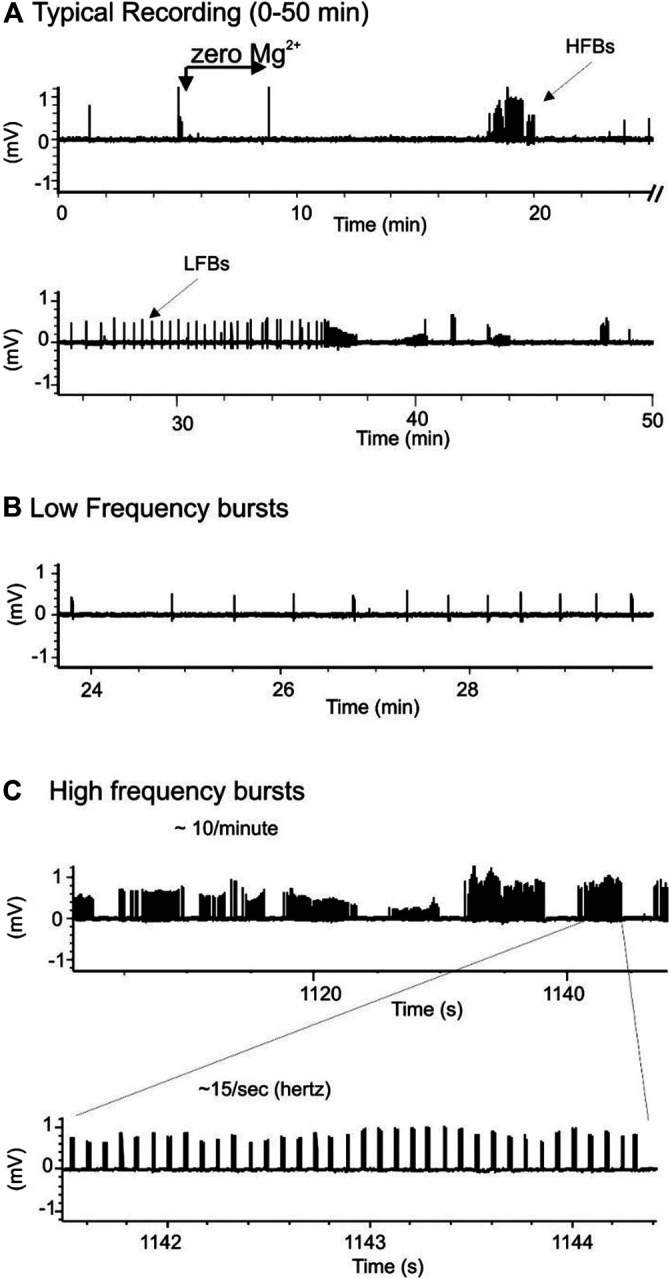
Chemically-induced seizure model produces two types of spontaneous response. **(A)**: Typical recordings of bursts in a hippocampal slice exposed to zero-Mg^2+^ high K^+^ medium. **(B,C)**: Expanded scales from A showing two types of spontaneous seizure activity induced by zero-Mg^2+^ high K^+^ medium: low frequency burst (LFB)-**B** and high frequency bursts (HFB)-C.

**FIGURE 8:**
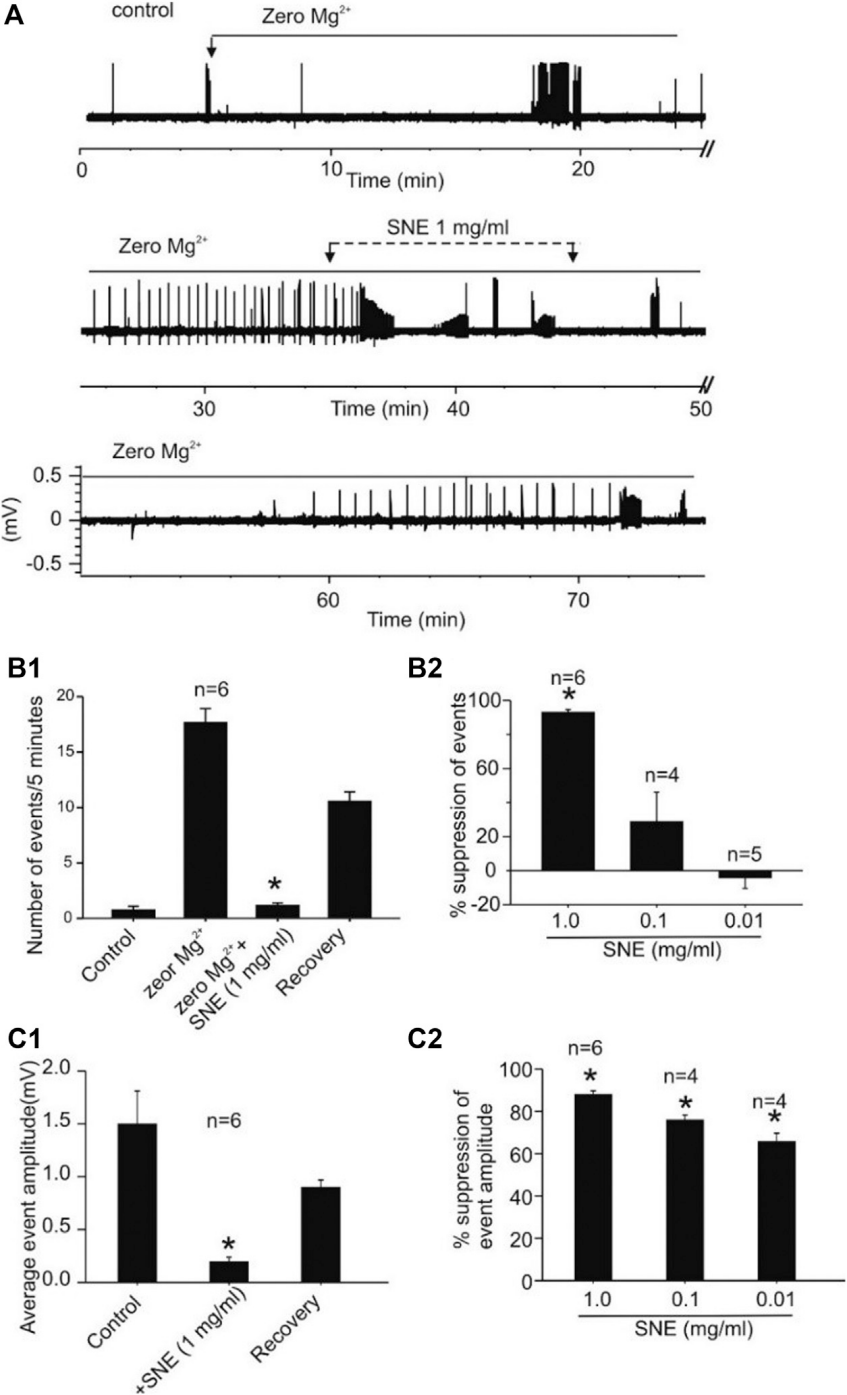
SNE suppresses the frequency and amplitude of spontaneous seizure activity induced by zero-Mg^2+^high K^+^ medium. **(A)**: Trace showing effect of SNE on chemically induced seizure activity. **(B1)**: Summary bar graph of effect of SNE (1 mg/ml) on the frequency of spontaneous activity. **(B2)**: Summary bar graph showing concentration dependent effect of SNE on seizure activity. **(C1)**: Summary bar graph of effect of SNE (1 mg/ml) on the amplitude of spontaneous events. **(C2)**: Summary bar graph showing a concentration-dependent depression of event amplitude by SNE.

When seizures were induced in slices using a modified aCSF that contained zero Mg^2+^ and high K^+^, two types of spontaneous activities were observed, low frequency burst (LFBs) and high frequency bursts (HFBs; Figure 7). Similar to the effect on evoked fEPSPs, increasing concentrations of SNE (0.01, 0.1, and 1.0 mg/ml) depressed the seizure activity in a concentration-dependent manner (Figures 8A,B1). SNE (0.1 and 1 mg/ml) decreased the frequency of firing of both LFBs and HFBs (*p* < 0.05) while 0.01 mg/ml had no effect (Figure 8B2; *p* > 0.05). By contrast, SNE at all doses tested significantly depressed the amplitude of both spontaneous events (Figure 8C1,C2; *p* < 0.05).

## Discussion

### Resource Identification Initiative

This study showed that SNE had the ability to suppress excitatiory synaptic activity in the hippocampus, a brain region implicated in the pathophysiology of certain seizures (Parent et al., 1997; Gunn and Baram, 2017; Cavarsan et al., 2018; Farrell et al., 2019; Farrell et al., 2019). This effect was concentration-dependent with relatively high potency and was not completely reversed after 20 min of washing out of the extract. The SNE-induced synaptic depression was accompanied by an increase in paired pulse facilitation suggesting the involvment of presynaptic mechanisms in the depression such as decreased neurotransmitter release. Two well established endogenous presynaptic modulators are excitation in the hippocampus GABA and adenosine (Xia et al., 2012; Real et al., 2018). In this regards, the SNE-induced synaptic depression was not mediated by adenosine through its A_1_ receptors as this effect was neither blocked nor reversed by the A_1_ receptor antagonist 8-CPT. Furthermore, SNE suppressed *in vitro* seizure activities induced by enhancing glutamate-mediated transmission by removal of Mg^2+^ and increasing K^+^, ions that play important roles in the excitability of neurons (Horne et al., 1986; Traynelis and Dingledine, 1988; Ananthalakshmi et al., 2007; Kombian and Phillips, 2013). The removal of Mg^2+^ and the depolarization caused by raising extracellular K^+^ combined to produce a hyperexcitatory state in hippocampal neurons due to the removal of voltage-dependent Mg^2+^ block of the NMDA receptors (Mayer et al., 1984; Kim and Robinson, 2011; Clarke et al., 2013; Huang and Gibb, 2014). The oscillatory discharge of neurons resulting from the above intervention is thought to be driven by changes in intracellular calcium (Mangan and Kapur, 2004; Gao and Goldman-Rakic, 2006). Indeed, a decrease in the availability of calcium pre-synaptically has the potential to decrease release probability, leading to an enhanced paired pulse facilitation as witnessed here. Thus, SNE may act at one or more targets to produce the reduction in epileptiform activity of the hippocampal neurons. These targets include NMDA and non-NMDA receptors, the intracellular calcium storage sites, or on voltage-gated sodium, potassium and calcium channels that ultimately drive action potentials and bursting behavior in neurons (Rogawski et al., 2016). It is well established that neuronal excitability is a delicate balance between glutamate driven excitation and GABA-dependent inhibition (Petroff, 2002; Calfa et al., 2015; Soukupova et al., 2015; Page and Coutellier, 2019). It is thus, also possible that SNE acted to enhance GABAergic inhibition to counterbalance the glutamate-induced hyper-excitation in this model. This may be through one of several mechanisms including, release of GABA (Rowley et al., 2012; Rassner et al., 2016), blockade of GABA reuptake (Rowley, et al., 2012; Rogawski et al., 2016), inhibition of GABA metabolizing enzymes (Treiman, 2001; Silverman, 2018) or direct interaction with GABA receptors and their channels (Sperk et al., 2004; Mareš and Kubová, 2015; Avoli, 2019). All these possibilities need to be tested in subsequent studies to complete our understanding of how this extract produces its *in vitro* anti-seizure effect. Further to this, other potential targets of SNE to produce its anti-seizure effect may include adenosinergic systems (Mareš and Kubova, 2008; Alves et al., 2018). However, this is unlikely as the depressant effect of SNE on fEPSP were not affected by 8-CPT, an adenosine receptor A_1_ receptor antagonist, ruling out presynaptic adenosine A_1_ receptors.

SNE in this study appeared to be a very efficacious and potent suppressor of excitatory synaptic transmission given that as little as 1 mg/ml reduced the evoked fEPSP by nearly 80%, requiring only a fraction of that to reduce it by 50% (estimated IC_50_ = 0.06 mg/ml). This fEPSP effect of SNE was still significantly present after 20 min washing out of SNE suggesting that the effect can be relatively long lasting which may reflect a slowly reversible process of the components of the extract. Similar to its effect on the evoked fEPSPs, SNE also suppressed spontaneous bursting (both LFBs and HFBs) activity in hippocampal slices caused by the removal of Mg^2+^ that led to a glutamate driven hyperexcitability state. This effect was also concentration-dependent, resulting in a significant uppression in the occurrence of bursting and decreasing the amplitude of remaining bursts. A signifcant proportion of this depression was still present after 20 min washing out of SNE. This suggests that SNE may produce pharmacokinetically relevant suppression of seizure activity *in vivo,* confirming the earlier reported anti-seizure effects *in vivo* (Amoateng et al., 2012).

## Conclusion

This preliminary study has provided cellular evidence in support of the use of this herbal product in traditional practice to manage convulsions. It still remains to be determined the exact mechanism by which SNE does this and the active components that may be responsible for the neuroactivity of SNE. Followup studies are being conducted to isolate and purify the active ingredient(s) in SNE and to investigate the detailed cellular and molecular mechanism underlying SNE actions.

## Data Availability

The raw data supporting the conclusions of this article will be made available by the authors, without undue reservation.
